# Improved PEDOT:PSS/c-Si hybrid solar cell using inverted structure and effective passivation

**DOI:** 10.1038/srep35091

**Published:** 2016-10-11

**Authors:** Xisheng Zhang, Dong Yang, Zhou Yang, Xiaojia Guo, Bin Liu, Xiaodong Ren, Shengzhong (Frank) Liu

**Affiliations:** 1 Key Laboratory of Applied Surface and Colloid Chemistry, National Ministry of Education; Shaanxi Engineering Lab for Advanced Energy Technology; School of Materials Science & Engineering, Shaanxi Normal University, Xi’an, 710119, P. R. China; 2 Department of Physics and Electronic Engineering, Yuncheng University, Yuncheng University, Yuncheng, 044000, P. R. China; 3 Dalian Institute of Chemical Physics, Dalian National Laboratory for Clean Energy, Chinese Academy of Sciences, Dalian, 116023, P. R. China

## Abstract

The PEDOT:PSS is often used as the window layer in the normal structured PEDOT:PSS/c-Si hybrid solar cell (HSC), leading to significantly reduced response, especially in red and near-infrared region. By depositing the PEDOT:PSS on the rear side of the c-Si wafer, we developed an inverted structured HSC with much higher solar cell response in the red and near-infrared spectrum. Passivating the other side with hydrogenated amorphous silicon (a-Si:H) before electrode deposition, the minority carrier lifetime has been significantly increased and the power conversion efficiency (PCE) of the inverted HSC is improved to as high as 16.1% with an open-circuit voltage (V_oc_) of 634 mV, fill factor (FF) of 70.5%, and short-circuit current density (J_sc_) of 36.2 mA cm^−2^, an improvement of 33% over the control device. The improvements are ascribed to inverted configuration and a-Si:H passivation, which can increase photon carrier generation and reduce carrier recombination, respectively. Both of them will benefit the photovoltaic performance and should be considered as effective design strategies to improve the performance of organic/c-Si HSCs.

Compared with traditional high temperature p-n junction fabrication, the organic/c-Si hybrid solar cells (HSC), which show advantages in economic processing^1^,^2^,^3^,^4^,^5^,^6^,^7^,^8^,^9^,^10^,^11^, have attracted significant attention in recent years. The crystalline Si is used as the effective light absorbing material, while the organic film mainly functions as the hole transport layer in addition to its antireflection role^12^,^13^,^14^. The most common organic materials developed so far include poly (3-hexylthiophene), (P3HT) 2,2′,7,7′-tetrakis-[N,N-di(4-methoxyphenyl]amino]-9,9′-spirobifluorene(spiro-OMeTAD), ploy (3,4-ethylene dioxythiophene):poly-strenesulfonate (PEDOT:PSS) and so on^15^,^16^,^17^,^18^. Among these, PEDOT:PSS is the most favorite one as the hole transport material (HTM) on c-Si for its high transparency, hole-conducting ability, suitable work function, and easy fabrication methods^19^,^20^,^21^. Over the past few years, the reported efficiency of c-Si HSC based on PEDOT:PSS is mostly around 13%^5^,^13^,^19^,^22^,^23^. In these normal hybrid solar cells (HSC), the PEDOT:PSS is often used as the window layer, which lead to significantly reduced response, especially in red and near-infrared region^22^,^24^,^25^,^26^,^27^, due to parasite absorption of PEDOT:PSS film, as reported by Dimitri Zielke *et al*.^28^. In addition, the opposite side of c-Si surface of these cells is poorly passivated and directly contacted with rear metal electrode, leading to low V_oc_ deriving from the serious recombination losses.

In present work, we develop the inverted structured HSC with configuration of ITO/a-Si:H/c-Si/PEDOT/Ag, which combine advantages of transparent electrode and surface passivation of HIT solar cell as well as the back PEDOT:PSS electrode to improve the light utilization, charge collection efficiency and stability^29^. Intrinsic a-Si:H layer was used as passivation layer and highly doped n-type a-Si:H was deposited as a front surface field (FSF). All the work can be completed under 220 °C. Compared to the normal structured HSC with PEDOT:PSS deposited on the front of c-Si wafer surface as a window layer, the inverted structure design can lead to higher transmittance and lower reflection, which result in higher EQE in 500–1200 nm range and larger J_sc_ 36.2 mA cm^−2^. Meanwhile, a thin layer of a-Si:H is employed as the passivation layer onto the c-Si surface, which can increase the minority carrier lifetime and improve the V_oc_ of HSC from 548 mV to 634 mV. As a result, the power conversion efficiency (PCE) of the inverted HSC can reach as high as 16.2%, an improvement of 33% over the control device. Further device analysis based on carrier lifetime measurement show that the PCE of the inverted PEDOT:PSS/c-Si HSC can potentially exceed 20%.

## Results and Discussion

**Figure 1:**
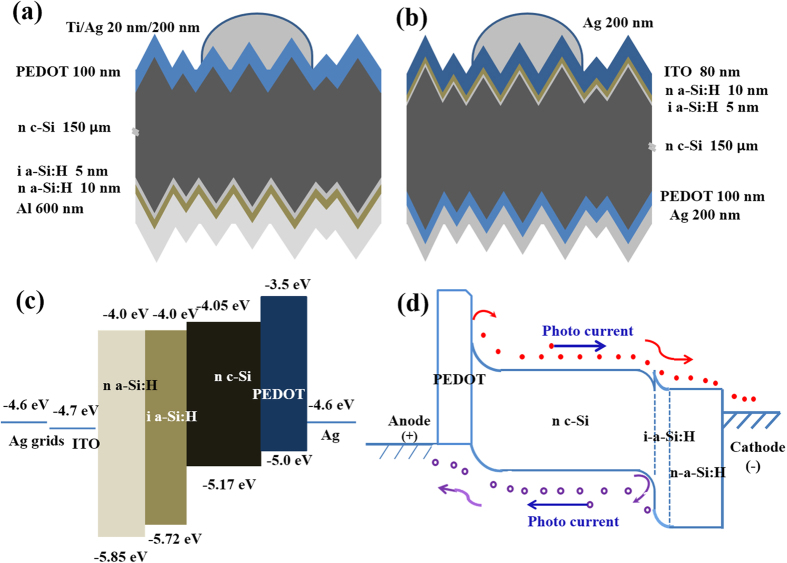
Schematics of (**a**) normal PEDOT:PSS/c-Si HSC and (**b**) inverted PEDOT:PSS/c-Si HSC combined with the intrinsic and n-type a-Si:H layer. (**c**) Energy band alignment of the inverted PEDOT:PSS/c-Si HSC. (**d**) Band diagram and carrier transport process under illumination.

To investigate the effects of a-Si:H passivation and back PEDOT:PSS on the performance of HSC, two modified HSCs have been proposed and fabricated as shown in Fig. 1a,b and listed in Table 1. Figure 1c,d presents the energy level diagram and carrier transport process in the Grid Ag/ITO/a-Si:H/c-Si/PEDOT/Ag solar cell. As seen in Fig. 1c, the levels of PEDOT are well aligned with the bands of n-type c-Si, which allows holes in silicon to flow into PEDOT unimpeded and reflect electron back into the bulk and reduce the surface recombination (Fig. 1d). Additionally, the insertion of n-type a-Si:H on the cathode electrode not only decreased the contact resistance, but also formed a electric field, which can facilitate electron collection as well as repel the hole into the bulk to reduce surface recomnication. These characters of this device configuration could increase photogenerated carriers collection efficiency and reduce dark current or J_0_ and carrier recombination, which leads to higher V_OC_.

**Figure 2:**
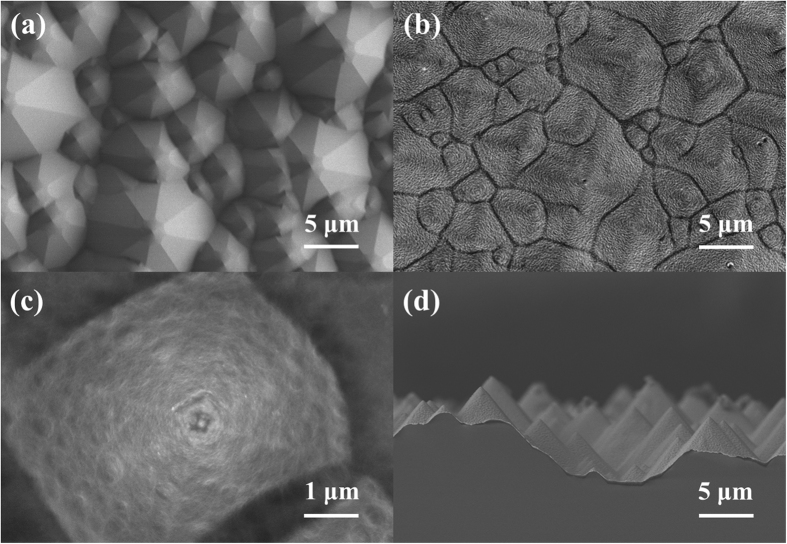
(**a**) The top-view SEM image of the pyramid c-Si surface. (**b**) The top-view SEM image of pyramid c-Si surface covered with PEDOT:PSS. (**c**) The high resolution SEM of pyramid c-Si surface covered by PEDOT:PSS. (**d**) Cross-sectional SEM image of pyramid c-Si surface covered by PEDOT:PSS.

The PEDOT:PSS/c-Si interface plays a key role in the cell performance^9^,^12^,^15^. Figure 2a,b show the top-view SEM images of pyramids structured c-Si wafer surface with and without PEDOT:PSS, respectively. It is seen that the bare c-Si wafer surface consists of pyramids with different size in the range of 5~10 μm. When the PEDOT:PSS infiltrates the gaps between the pyramids, it forms a uniform layer above the textured Si wafers. It is clearly seen that the c-Si pyramids are completely coated by PEDOT:PSS (Fig. 2c). Figure 2d shows cross-sectional image of the pyramid surface covered by the PEDOT:PSS layer. The hills and valleys of the Si pyramids are fully covered with about 100 nm PEDOT:PSS. The better coverage on the valleys is due to chemical etching, the larger pyramid size and better adhesion to hydrophobic c-Si surface comparing to the poorer coverage reported^23^. The good coverage with PEDOT:PSS coating is of benefit to reduce the surface recombination and charge collection^30^.

**Figure 3:**
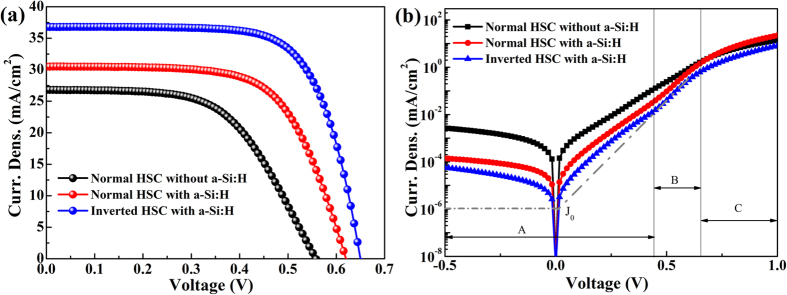
(**a**) J-V curves of both normal and inverted PEDOT:PSS/c-Si HSC. (**b**) Dark J-V characteristics of PEDOT:PSS/c-Si HSC.

To investigate the effect of a-Si:H on the surface passivation, a normal HSC with an a-Si:H passivation layer has been prepared. In Fig. 3a, the J-V curve of the champion PEDOT:PSS/c-Si HSC with and without the a-Si:H layer has been compared, with key J-V parameters listed in Table 2. It shows that the best cell without the a-Si:H film exhibits J_sc_ of 26.8 mA cm^−2^, V_oc_ of 548 mV and FF of 56.5%, yielding a PCE of 8.3%. The lower V_oc_ derives from the serious recombination losses at the fully metalized rear electrode, the direct contact between the metal electrode and the doped c-Si apparently forms a Schottky-barrier, as commonly observed at the metal-semiconductor interface^31^, leading to high contact resistance and thus an inferior FF and J_sc_. When the c-Si rear surface is passivated by intrinsic a-Si:H film and back surface field is constructed using highly doped n-type a-Si:H layer, the PCE of normal HSC is improved to 12.1% with J_sc_ of 29.7 mA cm^−2^, V_oc_ of 620 mV and FF of 65.8%. The substantial efficiency increase is attributed to the high quality passivation of the intrinsic a-Si:H thin film and n-type a-Si:H as back surface field. The intrinsic a-Si:H film is known to provide effective passivate to the surface defects between the c-Si and a-Si:H(n) layer, resulting in reduced recombination rate at the c-Si surface via a downward band bending for reflecting holes (minority carrier)^32^,^33^. In addition, the highly doped a-Si:H(n) film generates the strong built-in back surface electric field (BSF) and improves the electronic contact at the back surface, facilitating the separation and diffusion of the minority carriers at the front heterojunction, therefore significantly enhances the HSC cell performance.

As the a-Si:H could passivate the c-Si surface as well as form a transparent electrode with ITO, the inverted HSC with transparent ITO electrode and PEDOT:PSS back electrode has been fabricated and characterized. The ITO and a-Si:H thin film serves as window layer for the inverted PEDOT:PSS/c-Si HSC, the intrinsic a-Si:H is employed as the passivation layer, and the highly doped n-type a-Si:H forms a front surface field. Figure 3a compared the J-V curves of the best normal and inverted types of PEDOT:PSS/c-Si HSC passivated using the a-Si:H thin films. Surprisingly, the inverted PEDOT:PSS/c-Si device exhibits much better photovoltaic performance with J_sc_ 36.2 mA cm^−2^, V_oc_ 634 mV and FF 70.2%, giving a PCE of 16.1%. It is obvious that all key parameters are dramatically increased for the inverted HSC with PCE increased by as much as 33% compared to the normal-type HSC. *In addition, as the PEDOT:PSS is hygroscopic in nature, it may result in degradation of the HSC performance*^23^*. However, in the inverted HSC, the PEDOT:PSS layer is fully covered by the metal electrode, isolating it from direct contact with air. Therefore, it may make the inverted HSC more stable than the normal structured HSC*.

The dark J-V characteristic is often a good indicator for the solar cell performance. Figure 3b shows the *J-V* curves measured in dark for the normal HSC with and without a-Si:H passivation layers to compare with the inverted HSC. It is known that to attain a high V_oc_, the reverse saturation current density J_0_ must be reduced because the leakage current leads to reduced V_oc_. In theory, the dark J-V curves can be simulated using the diode Equation (1) 34


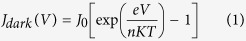


where n the diode ideality factor, k the Boltzmann constant, T the absolute temperature and e is charge of an electron. Figure 3b shows J_dark_-V curves of the normal HSC with and without the a-Si:H passivation layers compared with the inverted HSC measured in the dark. The J_dark_-V response in region A is affected by the shunt behavior, while the voltage ranges B and C rely more on exponential diode behavior and the series resistance, respectively. The reverse saturation current density J_0_ and the diode ideality factor (n) can be determined using numerical fit to the J_dark_-V curve in the region B. Based on the least square fitting of the J_dark_-V characteristic curves, the diode ideality factor (n) and reverse saturation current density (J_0_) of the PEDOT:PSS/c-Si heterojunction solar cells are extracted, as summarized in Table 3. The n values of the normal HSC without a-Si:H passivation layer are higher than what with passivation, which is most likely attributed to the injection-dependent recombination at the entirely metalized contact. The inverted HSC with a-Si:H solar cell shows the smallest n value, indicating the best passivation quality of the a-Si:H layer with good p-n junction. In additional, the J_0_ values displayed a similar tendency with the inverted HSC showing the smallest J_0_ value and the highest V_oc_.

**Figure 4:**
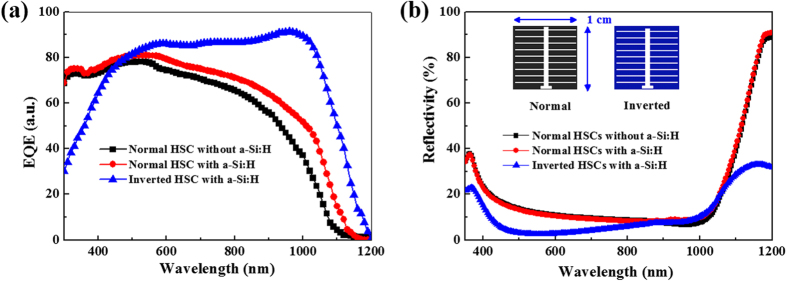
(**a**) EQE characteristics of the normal and inverted HSC. (**b**) Reflectance spectra of normal and inverted HSCs with bus/finger bars.

Figure 4a shows the external quantum efficiency (EQE) characteristics of the HSC including both normal and inverted structures with and without the a-Si:H passivation. The J_sc_ was confirmed by integrating the EQE with a standard AM 1.5 spectrum. The EQE integrated J_sc_ of the normal HSC with the a-Si:H layers increased by 2.9 mA cm^−2^ compared with the normal device without a-Si:H. The high EQE and J_sc_ are ascribed to higher passivation quality using the intrinsic a-Si:H and n-type a-Si:H as BSF, leading to reduced recombination at the back surface and enhanced carrier collection. Meanwhile, EQE of the inverted HSC with a-Si:H layers is significantly higher than that of normal device. It is attributed to the EQE gain from increased light trapping in spectrum 500–1200 nm. Figure 4b shows the reflectivity spectra of the HSC. It is clear that the reflectivity of the inverted HSC is lower than that of the normal device in 400–800 nm region because of the 80 nm thick ITO film in the inverted device serving not only as a transparent conductive coating but also an antireflection layer for improved light utilization. *However, the EQE below 500* *nm is low due to the absorption of a-Si:H. We therefore control the bilayer thickness to <15* *nm. As the photon flux in this range is far lower than that in the red spectrum, even though the EQE below 500* *nm is low, its contribution to J*_*sc*_ *is very little as shown in* Figure S2.

The reflectivity spectra of all three samples are similar in 800–1100 region. However, there are huge differences in EQE results among inverted and normal structured HSCs, which should be ascribed to the parasitic absorption in PEDOT:PSS layer reducing the photo response in red and NIR region. *As the photon flux at 600–1000* *nm in the AM1.5 spectrum is more abundant than in the UV region, the EQE enhancement to J*_*sc*_ *in the long wavelength range is more pronounced, as seen in* Figure S2. The EQE results clearly disclose the advantage of inverted HSCs in red and NIR photon utilization, which can effectively increase the photocurrent.

**Figure 5:**
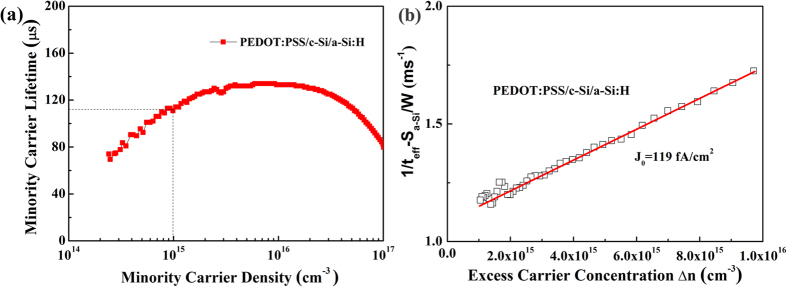
(**a**) Minority carrier lifetime of c-Si passivated with PEDOT:PSS and a-Si:H. (**b**) Measured inverse effective lifetime 1/τ_eff_ minus the surface-related lifetime S_a-Si:H_/W as a function of the excess carrier concentration Δn for c-Si passivated with PEDOT:PSS and a-Si:H.

To elucidate the potential advantages of passivation of the c-Si using a-Si:H and PEDOT:PSS, we measured minority carrier lifetime (MCL) with transient photoconductance decay (PCD) using a WCT-120 lifetime tester from Sinton Instruments^35^,^36^. The MCL is up to 112 μs for PEDOT:PSS/c-Si/a-Si:H heterojunctions at an injection level of 1 × 10^15^ cm^−3^, as seen in Fig. 5a, ten times larger than device without a-Si:H passivation. Based on the injection-dependent lifetime data, the reverse saturation current density J_0_ is calculated by fitting the reciprocal effective lifetime 1/τ_eff_ versus the excess carrier concentration Δn using the Equation (2)


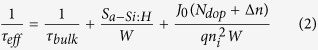


where W is the thickness of wafer, q the elementary charge, and n_i_ = 9.63 × 10^9^ cm^−3^ is the intrinsic carrier concentration of silicon at absolute temperature (298 K), N_dop_ = 3 × 10^15^ cm^−3^ 37. Note that the influence of bulk minority carrier lifetime (τ_bulk_) on the J_0_ value is negligible because the value of τ_bulk_ is much higher than τ_eff_. An effective surface recombination velocity S_a-Si:H_ is measured in the range between 3.5 and 7.5 cm/s (excess carrier density in the range between 10^14^ and 10^16^ cm^−3^) for the wafer passivated with a-Si:H, *as shown in supporting information* (Figure S3 and Table S1) Fig. 5b shows the J_0_-related contribution of Eq. (2) as a function of excess carrier concentration Δn in the silicon sample. A low saturation current density J_0_ of only 119 fA cm^−2^ was extracted according to a linear fit of Eq. (2) to the measured data.

It is known that V_oc_ is determined by the reverse saturation current density (J_0_) and J_sc_^38^,^39^, as Equation (3)


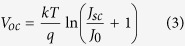


where kT/q = 25.69 mV at absolute temperature (298 K). J_sc_ is about 40 mA cm^−2^ for c-Si solar cells with high efficiency^40^. Based on Equation (3) and the experiment results of J_o_ shown in Fig. 5, we calculated implied V_oc_ value. The implied V_oc_ is 682 mV, respectable value for this type of solar cells. It is worthwhile to point out that these results are consistent with the previous V_oc_ measurements with good FF at about 70%. With further improvement in the PEDOT:PSS/c-Si interface passivation, it is expected that higher PCE of the inverted HSC can be achieved by improving the V_oc_ and FF.

## Conclusion

In conclusion, we implanted the intrinsic and n-type a-Si:H layers to the rear surface of normal PEDOT:PSS/c-Si HSC, which can reduce the carrier recombination rate and increase the PCE from 8.3% to 12.1%. The PCE is further improved to 16.1% using the inverted structure, in which the parasitic absorption of PEDOT:PSS has been eliminated, resulting in an improvement of 33% over the normal-type device. The improvement can be attributed to well-matched energy level, improved light utilization efficiency of inverted structure, and effective suppression of carrier recombination. Furthermore, the device stability has been prominently enhanced. It is expected that PCE of the inverted PEDOT:PSS/c-Si HSC can be further increased to 20% once the passivation is optimized.

## Methods

### Substrate Preparation

150 μm thick (100)-oriented Czochralski (CZ) n-type crystalline silicon wafers with resistivity of 1–3 Ω·cm were used as substrate. The c-Si wafers with area of 1 × 1 cm^2^ were sliced using laser cutting. The c-Si substrates were cleaned in acetic acid solution for 15 min, then the saw damage was removed by immersing them in 20 wt% potassium hydroxide solution for 60 s. The pyramid structure was obtained in 5 wt% potassium hydroxide and 10 wt% isopropyl alcohol solution for 20 min under 80 °C. Finally, after the textured wafers are etched in acid solution (95% HNO_3_ and 5% HF) at 8 °C for 80 s. they are cleaned with standard RCA method and dipped in 5% HF for 60 s to remove the surface native oxide layer.

### Device Fabrication

For device fabrication, following standard RCA cleaning and a dilute HF dip, 5 nm thick intrinsic hydrogenated amorphous silicon (a-Si:H(i)) film was deposited on one side of the processed wafers by plasma-enhanced chemical vapor deposition (PECVD, MV system, USA) to act as passivation layer. Surface field layer was realized by subsequent deposition of 10 nm thick highly doped a-Si:H(n). The high quality intrinsic layers were deposited at 220 °C by regulating the process gas ratio of SiH_4_ and H_2_, while n-type layers were at 200 °C with SiH_4_, H_2_, and PH_3_. For hole collection, a 100 nm PEDOT:PSS (Heraeus Precious Metals GmbH & Co, KG, Clevios PH 1000) layer was spin-coated on the opposite side of the wafer. The PEDOT:PSS solution was mixed by 5% dimethylsulfoxide (DMSO) and 0.5% Triton 100, and spin coated at 2000 revolutions per minute (rpm) for 10 s and subsequently 3000 rpm for 40 s, then dried on a hotplate at 150 °C for 15 min.

Noticeably, the HSC with PEDOT:PSS on the front as a window layer is defined as normal PEDOT:PSS/c-Si HSC. The schematic of normal PEDOT:PSS/c-Si HSC is shown in Fig. 1a. The finger grid of titanium (10 nm) and silver (200 nm) is thermally deposited as a top electrode (Fig. 1a) by a bus-finger mask. A 600 nm thick aluminum (Al) film was deposited on the a-Si:H layer along the whole back surface of the Si substrate as the back electrode. Oppositely, the inverted PEDOT:PSS/c-Si HSC with indium tin oxide (ITO) and a-Si:H as window layer is fabricated with PEDOT:PSS deposited on the rear c-Si wafer surface. The ITO is sputtered with CVD on the front and metalized with Ag finger grids. The schematic illustration of inverted PEDOT:PSS/c-Si HSC is shown in Fig. 1b. The structures of as-prepared HSCs are listed in Table 1.

### Measurements

The current density-voltage (J-V) characteristic was measured using a KEITHLEY 2400 source-measure unit both under dark and AM1.5G illumination at 100 mW cm^−2^. The J_sc_ was confirmed by the integrated product of the external quantum efficiency (EQE). The EQE was measured using a quantum efficiency measurement instrument (CROWNTECH, QTEST STATION 500TI). The UV-Vis-NIR diffuse reflection spectrum was measured using the SHIMADZU UV-3600 spectrophotometer. The scanning electron microscope (SEM) was performed on a JEOL JSM-6700F. WCT-120 lifetime tester from Sinton Instruments was applied for minority carrier lifetime test.

## Additional Information

**How to cite this article**: Zhang, X. *et al*. Improved PEDOT:PSS/c-Si hybrid solar cell using inverted structure and effective passivation. *Sci. Rep*. **6**, 35091; doi: 10.1038/srep35091 (2016).

## Supplementary Material

Supplementary Information

## Figures and Tables

**Table 1:** Structure design of the HSCs.

Device	Structure
Normal HSC w/o a-Si:H	Grid Ag/PEDOT/c-Si/Al
Normal HSC with a-Si:H	Grid Ag/PEDOT/c-Si/a-Si:H/Al
Inverted HSC with a-Si:H	Grid Ag/ITO/a-Si:H/c-Si/PEDOT/Ag

**Table 2:** Key J-V parameters of PEDOT:PSS/c-Si HSCs.

HSC		V_oc_ (mV)	J_sc_ (mA cm^−2^)	FF (%)	PCE (%)	R_s_ (Ω m^2^)	R_sh_ (kΩ cm^2^)
Normal w/o a-Si:H	average	538 ± 10	26.5 ± 0.5	55.7 ± 0.8	7.92 ± 0.39	13.79 ± 0.52	0.27 ± 0.07
	best	548	26.8	56.5	8.3	13.27	0.34
Normal with a-Si:H	average	615 ± 5	29.5 ± 0.3	65.5 ± 0.4	11.84 ± 0.29	11.88 ± 0.37	1.86 ± 0.05
	best	620	29.7	65.8	12.1	11.51	1.91
Inverted with a-Si:H	average	630 ± 4	36.0 ± 0.3	70.3 ± 0.3	15.87 ± 0.25	10.30 ± 0.24	3.26 ± 0.02
	best	634	36.2	70.5	16.1	10.06	3.28

**Table 3:** Diode ideality factor (n) and reverse saturation current density (J_0_) of the normal and inverted HSCs.

HSC	n	J_0_ (mA cm^−2^)
Normal w/o a-Si:H	2.5	2.82*10^−4^
Normal with a-Si:H	1.9	1.23*10^−5^
Inverted with a-Si:H	1.7	1.03*10^−6^
