# Supplementary material for: Improved PEDOT:PSS/c-Si hybrid solar cell using inverted structure and effective passivation
Source: Sci Rep. 2016 Oct 11;6:35091. doi: 10.1038/srep35091 (PMC5057131; doi:10.1038/srep35091)
Supplement: Supplementary Information [file srep35091-s1.pdf]

Improved PEDOT:PSS/c-Si hybrid solar cell using inverted structure and effective passivation

Xisheng Zhang[1], Dong Yang[1,*], Zhou Yang[1], Xiaojia Guo[1], Bin Liu[1], Xiaodong Ren[1], Shengzhong (Frank) Liu[1,2,*]

[1]Key Laboratory of Applied Surface and Colloid Chemistry, National Ministry of Education; Shaanxi Engineering Lab for Advanced Energy Technology; School of Materials Science & Engineering, Shaanxi Normal University, Xi'an, 710119, P. R. China

[2]Dalian Institute of Chemical Physics, Dalian National Laboratory for Clean Energy, Chinese Academy of Sciences, Dalian, 116023, P. R. China

[*]**Corresponding author**: szliu@dicp.ac.cn; dongyang@snnu.edu.cn.

Figure Captions

Figure S1. The PCE stability of three types of hybrid solar cells stored in ambient conditions.

Figure S2. EQE and integrated J_{sc} of normal and inverted PEDOT:PSS/c-Si HSC.

Figure S3. The minority carrier lifetime of c-Si passivated with bilayer a-Si:H (i 5 nm and n 10 nm) **(a)** and PEDOT:PSS (100 nm) **(b).**

Figure S1

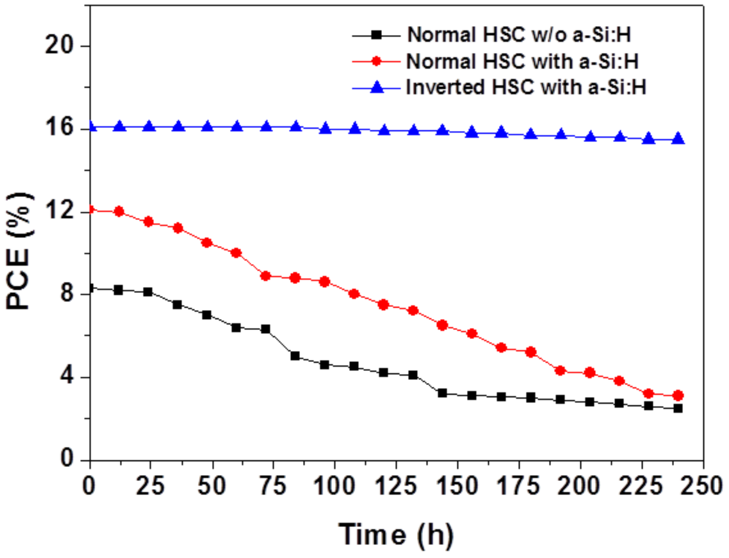

Figure S2

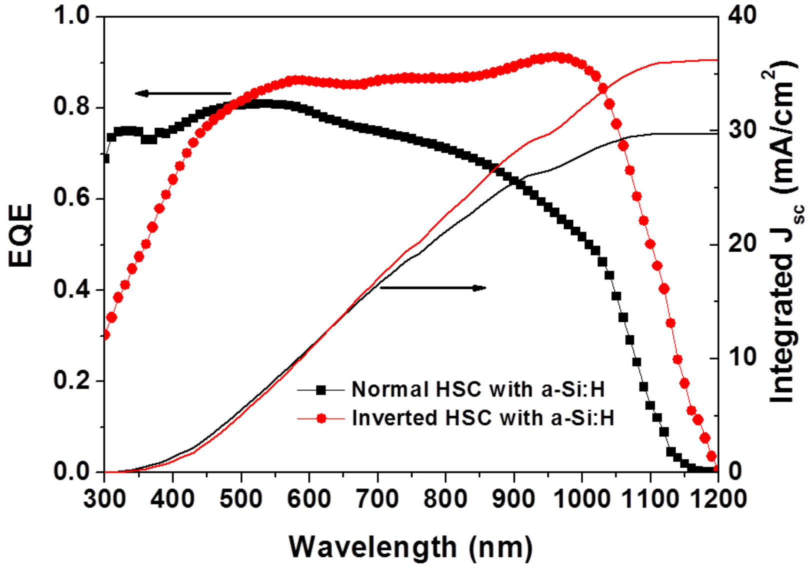

Figure S3

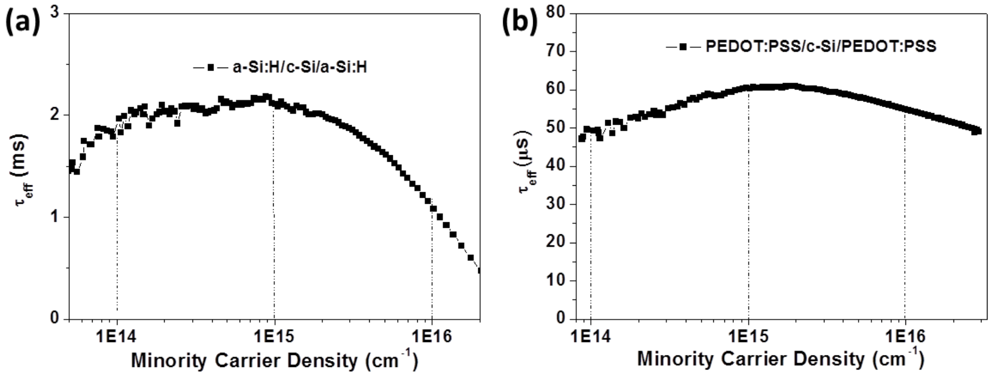

Table S1 The minority carrier lifetime (τ_{eff}) and effective surface recombination velocity S_{eff} at different injection minority carrier density (MCD).

Type \ MCD (cm^{-1})		1×10^{14}	1×10^{15}	1×10^{16}
a-Si:H/c-Si/ a-Si:H	τ_{eff} (ms)	1.96	2.08	1.05
	S_{eff} (cm/s)	7.4	7.5	3.5
PEDOT/c-Si/PEDOT	τ_{eff} (μs)	49.3	60.3	54.7
	S_{eff} (cm/s)	151	124	137
